# UP-REGULATION OF LONG NON-CODING RNA MORBID ATTENUATES SENESCENCE

**DOI:** 10.1093/geroni/igz038.344

**Published:** 2019-11-08

**Authors:** Zheng Kuai, Meiting Chen, yang yu, Fan Yang, chunxiang Zhang

**Affiliations:** 1 Department of Geriatrics,Zhongshan Hospital, Fudan University, Shanghai, China; 2 the University of Alabama at Birmingham, Alabama, United States

## Abstract

Aging is the inevitable, irreversible decline in function on the cellular and organ level leading to increased incidence of the most frequent diseases such as cancer and cardiovascular disease, that occurs over time. whereas the molecular mechanisms of senescence remain largely unknown. Here we identified that a novel long noncoding RNA, Morrbid was significantly decreased in different organs of aged mice, such as heart, liver, spleen, lung, kidney and brain. Interestingly, the telomeres length of Morrbid KO mice were significantly shorted than the WT mice at the same age. We also found that Morrbid was steeply decreased in a natural mouse cardiac myocyte senescence model. The senescence of mouse cardiac myocytes was effectively attenuated by Morrbid over-expression shown by the decreased β-galactosidase staining, increased telomere activity, decreased production of ROS and decreased cell apoptosis, but was enhanced by Morrbid knockdown. The results suggest that Morrbid is a critical regulator in senescence and could be used as a novel diagnostic biomarker for it, and a new therapeutic target for diverse diseases.

